# Toxoplasmosis seroprevalence in wild small rodents, potentially preys of ocelots in north-eastern Mexico

**DOI:** 10.1051/parasite/2014058

**Published:** 2014-11-07

**Authors:** Emilio Rendón-Franco, Lizbeth Xicoténcatl-García, Claudia Patricia Rico-Torres, Claudia Irais Muñoz-García, Arturo Caso-Aguilar, Gerardo Suzán, Dolores Correa, Heriberto Caballero-Ortega

**Affiliations:** 1 Departamento de Producción Agrícola y Animal, Universidad Autónoma Metropolitana – Unidad Xochimilco Mexico; 2 Laboratorio de Inmunología Experimental, Instituto Nacional de Pediatría, Secretaría de Salud México D.F. 04530 Mexico; 3 Caesar Kleberg Wildlife Research Institute, Texas A&M University-Kingsville 700 University Blvd., MSC 218 Kingsville TX 78363 USA; 4 Facultad de Medicina Veterinaria y Zootecnia, Universidad Nacional Autónoma de México México 04510 Mexico

**Keywords:** *Toxoplasma gondii*, Rodents, Seroprevalence, Molecular diagnosis, Mexico

## Abstract

The aim of this study was to assess the prevalence of *Toxoplasma gondii* infection in rodents that coexist with ocelots in north-eastern Mexico. Eighty rodents of five genera were captured and their serum samples tested for specific IgG antibodies to *T. gondii* by in-house indirect ELISA using three different conjugates. Prevalences of 7% (3/44) and 33% (4/12) were found in *Sigmodon hispidus* and *Liomys irroratus*, respectively, and were significantly different. All *Baiomys taylori* and *Oligoryzomys fulvescens* were negative for the presence of anti-*T. gondii* IgG antibodies. The samples from *Peromyscus* spp. could not be analyzed because none of the three conjugates tested recognized their immunoglobulins. Infection was confirmed in one single specimen of *L. irroratus* by qPCR, which generated an estimate of 146 parasites per mg of muscle tissue. The results strongly support the notion of active *T. gondii* transmission between rodents and felines in this zone of Mexico and an important role of some rodent species in the sylvatic cycle of *T. gondii*.

## Abstract (fr)

Le but de cette étude était d’estimer la prévalence de l’infection par *Toxoplasma gondii* chez les rongeurs qui coexistent avec des ocelots dans le nord-est du Mexique. Quatre-vingts rongeurs appartenant à cinq genres différents ont été capturés et les anticorps IgG spécifiques anti-*T. gondii* ont été recherchés dans leurs sera par une méthode ELISA indirecte développée dans le laboratoire, utilisant trois conjugués différents. Des prévalences de 7 % (3/44) et 33 % (4/12) ont été trouvées respectivement chez les espèces *Sigmodon hispidus* et *Liomys irroratus*, cette différence étant statistiquement significative. La recherche d’anticorps IgG anti-*T. gondii* a été négative chez tous les *Baiomys taylori* et *Oligoryzomys fulvescens*. Les sera des *Peromyscus* spp. n’ont pas été analysés car aucun des trois conjugués testés n’a reconnu leurs immunoglobulines. L’infection a été confirmée par qPCR chez un spécimen de *L. irroratus* avec une quantité de 146 parasites par mg de tissu musculaire. Ces résultats suggèrent fortement une transmission active de *T. gondii* entre rongeurs et félidés dans cette zone du Mexique et un rôle important de certaines espèces de rongeurs dans le cycle selvatique de *T. gondii*.

## Introduction

*Toxoplasma gondii* is an obligate intracellular parasite capable of infecting all warm-blooded animals including rodents, which are common intermediate hosts. Its definitive hosts are all members of the Felidae family, including the domestic cat (*Felis catus*) so there are domestic, synanthropic, and wildlife cycles [12]. Parasite dynamics are well understood in domestic animals and in humans, but little is known about the wild cycle [22]. The gaps in this knowledge are important in view of the increasing interaction between wildlife and urban populations and the risks for species conservation and public health.

Predation is a fundamental aspect of *T. gondii* transmission; consequently rodents are important sources of infection for domestic, feral, and wild cats. These small mammals compose two-thirds of the diet of wild small and medium felids, although this proportion may vary depending on several factors, including season, rodent density, felid species, and the presence of other prey [19].

Ocelots are medium-sized felids with a diet that mainly consists of vertebrates smaller than 1000 g [20]. In Mexico, De Villa et al. (2002) showed that rodents were the main components of their diet, *Liomys pictus* being the most frequent [9]. Although some authors have highlighted the theoretical role of rodents in the maintenance of *T. gondii* in wildlife, there are few reports of prevalence and distribution of this protozoan in wild felids [2]. A previous study in Soto La Marina, Tamaulipas, Mexico, demonstrated a prevalence of 69% of *T. gondii* infection in ocelots [21]. Although it is clear that these wild cats play an important role in the *Toxoplasma* wildlife cycle of this region, toxoplasmosis prevalence in the prey populations where these cats are the only possible definitive hosts is not known. In this study, we describe the prevalence of *T. gondii* infection using direct and indirect ELISA tests, as well as the parasite load estimated by qPCR, in rodents that coexist with ocelots in north-eastern Mexico.

## Material and methods

### Bioethics

Animals were trapped with authorization from the Mexican Ministry of Environment and Natural Resources (SEMARNAT), Registration No. FAUT-0250. The jurisdiction gives authorization to capture wild species for research purposes, provided the procedures do not cause harm.

### Rodent trapping

The study was performed at Los Ebanos ranch, located in Soto La Marina, Tamaulipas, Mexico, (24°30′ to 23°17′ N and –98°31′ to –97°44′ W) during September 2008. The predominant natural vegetation is tropical sub-deciduous forest, but pastures for livestock grazing exist as well. The area is on the coast of Tamaulipas, at 10 m above sea level. The weather at Soto La Marina is warm-moist, where temperature and rainfall ranges are 20–26 °C and 700–1100 mm, appropriate conditions for *T. gondii* transmission [16]. The study area was selected because two wildcat species have previously been recorded, jaguarundi (*Puma yagouaoroundi*) and ocelot (*Leopardus pardalis*), and a high prevalence of *T. gondii* infection was previously reported in the second species [5, 21].

All rodents trapped were included in the sampling protocol. They were captured using Sherman^®^ live traps (model 3310G, 10 × 3 × 3 inches, Tomahawk Live Trap Company, Tomahawk, Wisconsin, USA) baited with a mixture of peanut butter and oats. Quadrants (3600 m^2^) were established with 49 traps in each (in patterns of 7 × 7, 10 m apart from each other). The distance between the quadrants was around 500 m. Traps were set in a maximum of three quadrants (=147 traps/night) for two nights (total of 294 traps examined) up to complete nine quadrants (=882 traps). Each rodent trapped was handled according to the American Society of Mammalogists recommendations and identified using standard field guides [14]. A list of wild rodent species of the Tamaulipas State was created from the literature [17]. With this list and field guides, an identification key based on morphologic characteristics was developed and used in the field [14]. Body measurements were used for identification of each animal. The rodents were then bled by retro-orbital sinus puncture, and observed for at least 30 minutes (min) to check they were alert and responsive before releasing them. Blood samples were collected and centrifuged to obtain between 50 and 100 μL serum, which was aliquoted and frozen at −20 °C until tested. One *Liomys irroratus* specimen (Li62) died during manipulation, so samples from brain, lungs, skeletal muscles, liver, and kidney were collected and preserved for qPCR.

### Selection of conjugate by direct ELISA

Since there are no specific conjugates to detect immunoglobulins of wild rodents, three different conjugates coupled to peroxidase were tested by direct ELISA: Protein A (from *Staphylococcus aureus*), Protein G (from *Streptococcus pyogenes*), and anti-mouse IgG. A mixture of both proteins (PA + PG) was also tested. Polystyrene plates (Maxisorp Nunc, New York, USA) were sensitized with 100 μL/well of serum sample of each captured rodent from different genus, diluted 1:100 in 0.015 M carbonate buffer, pH 9.6 at 4 °C overnight. Plates were washed 5 times with 200 μL/well of 0.01 M phosphate buffered saline (PBS) with 0.05% Tween 20 (PBS-T) in an automated washer (BIO-RAD ImmunoWash 1575; Hercules, California, USA). Subsequently, 100 μL/well of Protein A (1:250), Protein G (1:250), a mixture PA + PG (1:250) or anti-mouse IgGγ chain specific (1:1000) developed in goat (Sigma-Aldrich Corp., St Louis, MO, USA, products P8170, P8651, and A3673, respectively) were added and incubated at 37 °C for 2 h. IgG was detected with 100 μL of a chromogen-substrate solution buffered with 0.1 M citrate (5 mL citric acid, 5 mL sodium citrate, 5 mg O-phenylenediamine-Sigma-Aldrich Life Science, St. Louis, MO, USA and 4.5 μL 30% hydrogen peroxide). The reaction was stopped with 50 μL/well 1 N sulphuric acid and absorbance values were measured at 490 nm in a Turner Biosystems 9300-010 Modulus Microplate Multimode Reader (CA, USA), using the Microplate Reader Modulus^TM^ software (Turner Biosystems, CA, USA).

### Detection of anti-*T. gondii* antibodies by indirect ELISA

Immunoassays were performed as previously standardized and taking into account the direct ELISA results [24]. Plates were coated with 100 μL/well of *T. gondii* crude extract from RH strain tachyzoites (2 μg/mL) in carbonate buffer at 4 °C overnight and washed as described above; next, non-specific binding sites were blocked with 200 μL of 1% bovine serum albumin (Euro-Clone, Milan, Italy, product JI001000173) for 30 min at 37 °C. Wells were washed and 100 μL of each serum sample, diluted 1:100, were added and incubated for 2 h at 37 °C. One hundred μL/well of Protein A (Sigma-Aldrich Corp.) diluted 1:250 were added and incubated at 37 °C for 2 h. After performing the appropriate washes, the antigen-antibody complexes were revealed and read as described above for direct ELISAs.

Because there were no wild rodent positive or negative control sera for detection of *T. gondii* infection, the cut-off point was calculated using the frequency distribution of the absorbance values, which had normal distribution, using their average plus 3 times the standard deviation, as previously described [23].

### Molecular diagnosis

*Toxoplasma gondii* DNA was detected using qPCR on the organs of one rodent of a single specimen of *L. irroratus* (Li62), which died during sampling. The parasite load was calculated in skeletal muscle using *B1* gene as a target, according to a previous report [6].

### Statistical analysis

Prevalences with 95% confidence intervals were calculated and the significance of differences among species was determined using Chi-square or Fisher’s exact tests, using Epidat 3.1^®^ software (Servicio de Epidemiología; Dirección Xeral de Innovación e Xestión de Saúde Pública, Santiago de Compostela, Coruña, Spain). Confirmation of the normal distribution of low absorbance values was determined by Shapiro-Wilk and Jarque-Bera tests.

## Results

**Figure 1.**
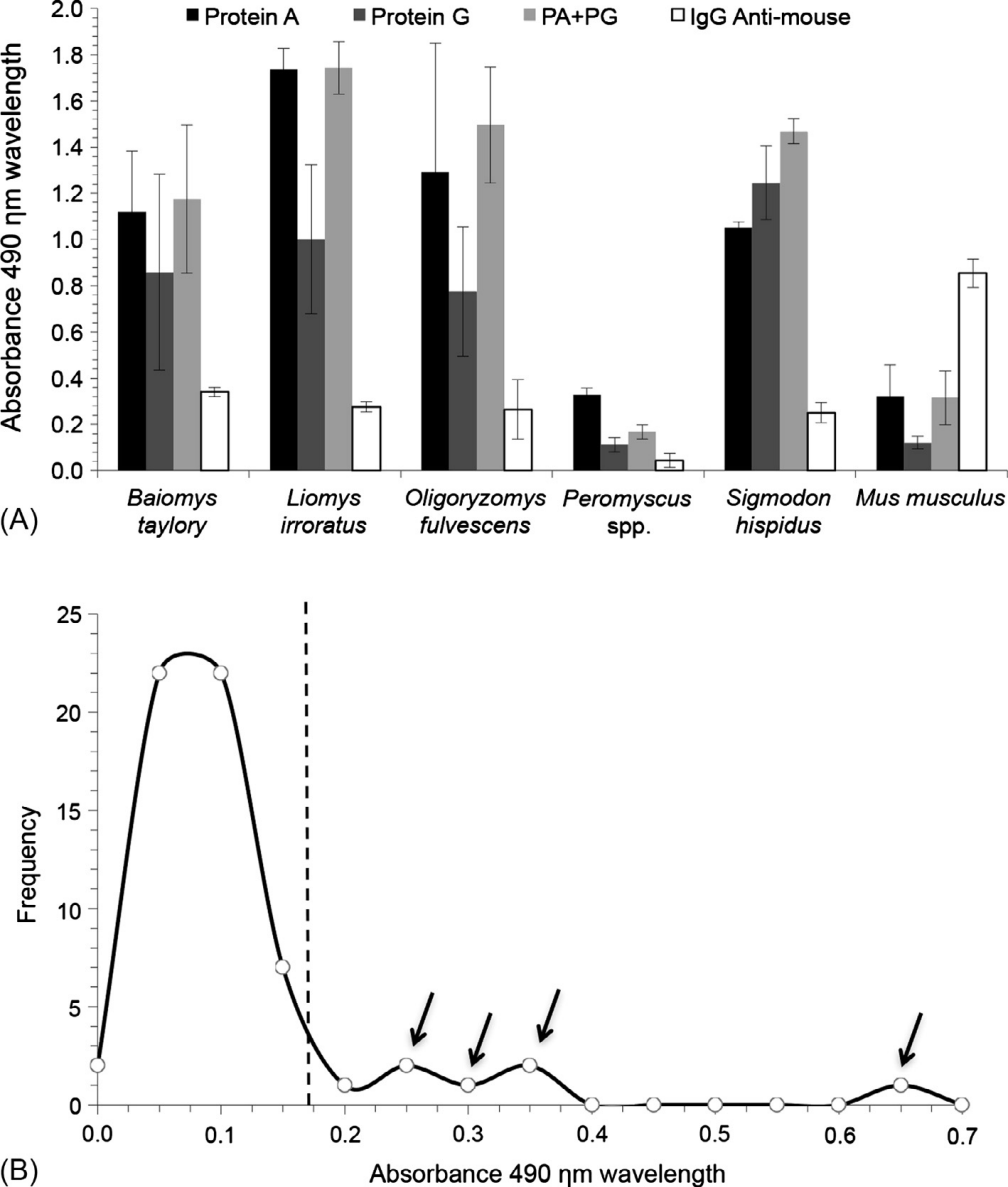
Immunoglobulin detection using three different conjugates and the mixture of Proteins A and G (PA + PG) by direct ELISA (A) and (B) frequency distribution of absorbance value in serum samples from four wild type rodents tested for anti-*T. gondii* antibodies by indirect ELISA. The dashed line is the calculated cut-off (Ab = 0.17). Normal distribution of negative sera was determined by Shapiro-Wilk (*P* = 0.421) and Jarque-Bera (*P* = 0.486) statistics. Arrows indicate positive samples as determined by their position to the right of the cut-off.

Eighty rodents of five genera were captured and sampled: *Baiomys taylori* (*n* = 2), *Oligoryzomys fulvescens* (*n* = 2), *Sigmodon hispidus* (*n* = 44), *Liomys irroratus* (*n* = 12), and *Peromyscus* spp (*n* = 20). Figure 1A shows that, with the exception of *S. hispidus*, the Protein A conjugate detected immunoglobulins (Igs) in all rodents efficiently. Samples from *Peromyscus* spp could not be analyzed because none of Protein A, Protein G nor anti-mouse IgG conjugates adequately recognized their Igs.

Normal distribution in low absorbance samples was demonstrated (*P* = 0.421 and *P* = 0.486 by Shapiro-Wilk and Jarque-Bera tests, respectively) and a cut-off point could be established. Few positive samples can be clearly seen at the right of this cut-off point (Figure 1B). The estimated prevalence of *T. gondii* was 6.8% (*n* = 44, CI 95% 1.43–18.66) and 33.3% (*n* = 12, CI 95% 9.93–65.11) for *S. hispidus* and *L. irroratus*, respectively; these values were significantly different (χ^2^ = 6.06, *P* = 0.013).

The DNA from *T. gondii* was detected in three (brain, skeletal muscles, and lungs) of five tissues of the *L. irroratus* specimen examined by qPCR. Due to the amount of available tissue and the quality of DNA extracted, quantification of the parasite load could only be performed in skeletal muscle. A mean load of 146 parasites per mg in this tissue was calculated; the correlation coefficient and E-value of the standard curve were 0.98 and 93.8% respectively, which reflected a very good linearity and high efficiency. Assuming that the parasite is homogeneously distributed, it is estimated that the rodent (about 48 g) may contain about 7 × 10^6^ parasites.

## Discussion

Microorganisms that cause zoonotic diseases, including those that are able to transit from wildlife to humans have public health implications. This is the case with *T. gondii*, which is common in domestic and wild animals [12]. Little is known, however, about the epidemiology of *T. gondii* in wildlife in Mexico. Dubey et al. (2009) demonstrated low seroprevalences of *T. gondii* in mice and rats (3.1% and 0.8%, respectively) and no seropositive terrestrial squirrels (*Spermophilus variegates*) from Durango in western Mexico [13]. Besides this, there are no studies on *T. gondii* prevalence in wild or synanthropic rodent species in Mexico. Here, we report the frequency of anti-*T. gondii* antibodies in wild rodents from Tamaulipas, in eastern Mexico. Since rodents are among the most species-rich groups, it is impractical to have specific conjugates for each genus or species. Thus, in this study an immunoassay to detect anti-*T. gondii* antibodies in different species of rodents was developed, using as conjugate protein A coupled to peroxidase [1]. It was adequate for all species tested except for *Peromyscus* spp. Thus, the prevalence of *T. gondii* in this rodent species could not be determined.

Prevalence of anti-*T. gondii* antibodies in *S. hispidus* was approximately 7%, which is among the lowest values reported in the literature [3, 7]. This host species is widely distributed and lives in quite diverse habitats. Therefore, it is likely that the large variation in the prevalence reported for this species could be due to environmental variations [4]. Although the role of *L. irroratus* in the epidemiology of *T. gondii* in wildlife in the area studied was suspected, the detection of antibodies reported here is the first record of the infection in these rodents, which are common preys of ocelots and was found in 62.7% of feline feces in Jalisco, Mexico [9]. It would be interesting to study the dynamics of *T. gondii* at different times of year in wild rodents, because a seasonal pattern of increase in transmission in autumn and winter and lower in spring and summer has been postulated. This seasonal increase in the proportion of infected rodents might lead to an increase in the risk of infection for felines [15].

Significant differences in *T. gondii* prevalence were observed between the two species of rodents that could be evaluated; this has been observed before both for wild and domestic hosts [18]. Ecological characteristics such as diet and longevity may explain these observations, as suggested by De Thoisy et al. (2003) [8]. Nevertheless, other authors have detected differences among rodent species, but no specific factors have been related to this variation [7]. Differences in the microhabitat should also be considered, since *L. irroratus* prefers steppe, bushes, and scrubland, while *S. hispidus* mainly eats pastures [4, 10].

In western Mexico, De Villa et al. (2002) reported that rodents were the main component of the ocelot diet, most notably *Liomys pictus*, which represented 24.4% of consumed prey [9]. This is in agreement with a preliminary work in which *T. gondii* seroprevalence of 69% was found in ocelots of the same region studied herein [21]. Although the previous result may seem incongruent with the low prevalence found in rodents, it is possible that low prevalence in prey may lead to high transmission if they are the main meat source for predators. As a matter of fact, one bradyzoite is enough to experimentally infect cats and the burden found in the mouse (146 parasite/mg tissue) strongly suggests transmission in the zone [11]. Nevertheless, it would be important to determine the prevalence in *Peromyscus* spp. since it might provide further data on *T. gondii* infection pressure to ocelots in the zone. A useful conjugate technique for this species and analysis of a larger number of specimens of the other two species would be useful to gather more information regarding relative importance of several rodents on *T. gondii* transmission. The MAT technique could not be used in this study mainly because of importation regulation problems during the study and the low amount of serum available after ELISA was performed. This was unfortunate because it might have been a good technique to estimate the prevalence in *Peromyscus* spp.

The identification of new “atypical” *T. gondii* variants, which cause disease in humans, domestic, and wild animals, has triggered concern from both biological and public health perspectives, because spatial overlap of the wild and urban cycles may represent pressure of disease occurrence [22]. Therefore, it would be important to characterize *T. gondii* strains circulating in wild, rural, and urban regions, which are close together.

In conclusion, *T. gondii* is prevalent in at least two endemic rodent species (*S. hispidus* and *L. irroratus*) of north-eastern Mexico where they are abundant and are preys of these felids. This report also expands the range of possibilities for serological diagnosis of *T. gondii* infection in some wild rodent species, using proteins A and G as conjugate in ELISA. So far, there are no reports of serological tests for detection of this protozoan in *Peromyscus* spp rodents; it would be interesting to test serum samples of this species with MAT. From the epidemiological viewpoint, it will be very interesting to analyze the genetic characteristics of strains of *T. gondii* that are circulating in wild animal populations and the degree to which sylvatic and domestic cycles are synonymous or distinct.

## Conflict of interest statement

The authors declare that they have no conflict of interest.
